# Novel functional insights into a modified sugar-binding protein from *Synechococcus* MITS9220

**DOI:** 10.1038/s41598-022-08459-8

**Published:** 2022-03-21

**Authors:** Benjamin A. Ford, Katharine A. Michie, Ian T. Paulsen, Bridget C. Mabbutt, Bhumika S. Shah

**Affiliations:** 1 School of Natural Sciences, Macquarie University, Sydney, Australia; 2 Mark Wainwright Analytical Centre, University of NSW, Sydney, Australia; 3 ARC Centre of Excellence in Synthetic Biology, Macquarie University, Sydney, Australia

**Keywords:** Biochemistry, Structural biology

## Abstract

Paradigms of metabolic strategies employed by photoautotrophic marine picocyanobacteria have been challenged in recent years. Based on genomic annotations, picocyanobacteria are predicted to assimilate organic nutrients via ATP-binding cassette importers, a process mediated by substrate-binding proteins. We report the functional characterisation of a *m*odified *s*ugar-*b*inding *p*rotein, MsBP, from a marine *Synechococcus* strain, MITS9220. Ligand screening of MsBP shows a specific affinity for zinc (K_*D*_ ~ 1.3 μM) and a preference for phosphate-modified sugars, such as fructose-1,6-biphosphate, in the presence of zinc (K_*D*_ ~ 5.8 μM). Our crystal structures of apo MsBP (no zinc or substrate-bound) and Zn-MsBP (with zinc-bound) show that the presence of zinc induces structural differences, leading to a partially-closed substrate-binding cavity. The Zn-MsBP structure also sequesters several sulphate ions from the crystallisation condition, including two in the binding cleft, appropriately placed to mimic the orientation of adducts of a biphosphate hexose. Combined with a previously unseen positively charged binding cleft in our two structures and our binding affinity data, these observations highlight novel molecular variations on the sugar-binding SBP scaffold. Our findings lend further evidence to a proposed sugar acquisition mechanism in picocyanobacteria alluding to a mixotrophic strategy within these ubiquitous photosynthetic bacteria.

## Introduction

Unicellular marine picocyanobacteria (*Prochlorococcus* and *Synechococcus*) are highly abundant primary producers, directly impacting global ocean ecosystems^1,2^. Marine picocyanobacteria are delineated into ecotypes based on conserved genetic markers correlating with discrete spatio-temporal distributions, environmental niches, or ocean depth^2,3^. Both marine *Synechococcus* and *Prochlorococcus* are exemplified by streamlined genomes (2.20–2.86 Mbp and 1.64–2.70 Mbp, respectively)^2^, reflecting intense environmental pressures to discard superfluous genes, retaining only the repertoire of genes required to survive in particular environments^4,5^. Strain-level heterogeneity in marine picocyanobacteria further indicates phyletic differences are highly correlated with environmental niches, and thus potentially define their metabolic strategies^6^.

Despite being regarded primarily as obligate photoautotrophs, metagenomic analyses predict marine picocyanobacteria harbour the genetic potential to uptake organic carbon^7–9^. Studies demonstrate differential ATP-dependent expression in glucose uptake and utilisation genes upon addition of glucose to laboratory cultures^10,11^, implying active transport. Marine picocyanobacteria apportion large parts of their transport capacity to proteins predicted to facilitate survival in nutrient-poor environments^12^, mainly via ATP-binding cassette (ABC) transport machinery, and are notable for possessing multiple substrate-binding proteins (SBPs) predicted to mediate uptake of organic and inorganic compounds^13^. These include predicted sugar-uptake proteins (*e.g.*, Cyanorak Clusters CK_1342 and CK_1455)^14^. The carbonate-binding protein, CmpA, from freshwater *Synechocystis* sp. PCC 6803 is the only cyanobacterial carbon-binding protein characterised to date^15^. CmpA binds inorganic C (C_*i*_) (CO_3_^2^ and HCO_3_^-^) in a pH-dependent manner and functions as part of an operon induced under low CO_2_ conditions^15^. To date, no picocyanobacterial SBPs predicted for sugar uptake have been experimentally characterised.

SBPs function primarily with ABC uptake machinery in bacteria and archaea to mediate nutrient recognition and translocation into the cell^16^. Numerous examples exist of the SBP scaffold being co-opted for use in other systems, *e.g*., two-component response regulation, chemotaxis, and protection against oxidative stress^16,17^. Recent evidence also indicates additional cryptic functionality, such as the recruitment of “orphan” SBPs in stress response mechanisms^18,19^ and nuanced control of transporter activity exerted by concomitant binding of metal ions^20^.

SBPs conform to a highly conserved structural architecture, comprising two Rossmann domains connected by a flexible hinge, enclosing a binding cleft embellished with molecular determinants suitable for interaction with their cognate chemical substrate^21,22^. A current SBP classification scheme^21^ defines seven broad SBP groups (A–G), some of which correlate with specific ligand chemistry (*e.g.* metal ions and oxoanions in group A). For other groups (e.g*.,* group D, whose ligands span sugars, sugar alcohols, amino acids and polyamines, inorganic polyanions, and metal ions), the very similar structural architectures require arbitrarily designating sub-clusters based on experimentally-determined substrate preference^22^.

The difficulty in predicting specific substrate chemistries and the potential for additional overarching regulation of cryptic function highlights the need for experimental validation to underpin functional designations of the SBP family^23,24^. This is especially true for those thought to be involved in non-canonical nutrient uptake, such as the annotated sugar-binding proteins from *Synechococcus* implied to contribute to a mixotrophic metabolic strategy^8^. We undertook large-scale ligand screening and solved two crystal structures of a predicted sugar-binding protein (hereafter referred to as MsBP, *m*odified sugar-*b*inding *p*rotein) from *Synechococcus* sp. MITS9220 (MITS9220_00117). Our structural results and investigation of ligand-binding capabilities highlight novel molecular variations on the sugar-binding SBP scaffold and reinforce the potential for mixotrophy in picocyanobacteria.

## Results and discussion

### Phylogenetic and genomic analysis of MsBP

**Figure 1:**
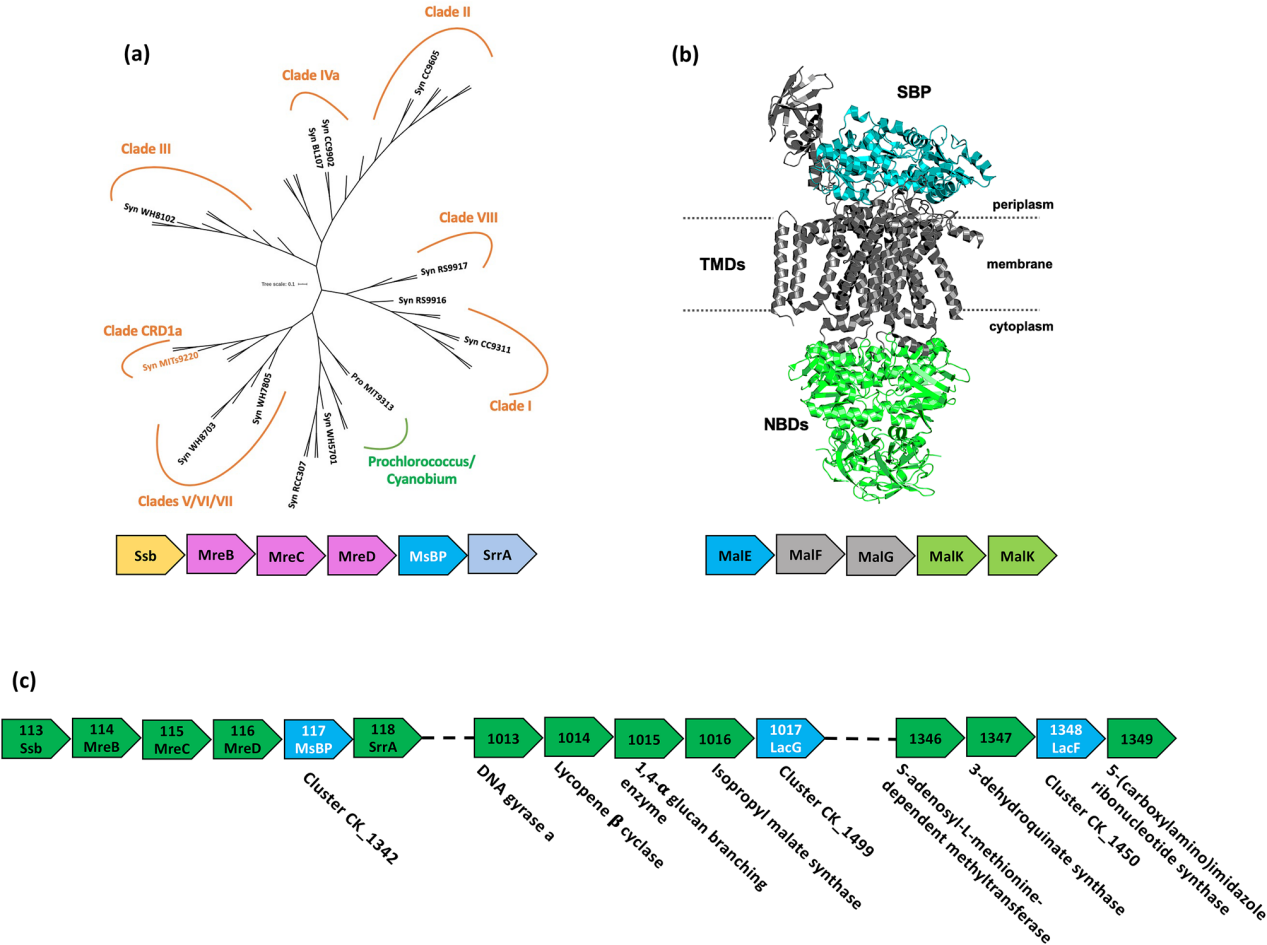
The phylogenetic and genomic context of MsBP **(a)** The phylogenetic tree for the conserved CK_1342 cluster is shown across *Synechococcus* clades, with 56 orthologous sequences (Cyanorak v2.1) The tree is labelled with the different clade-level classifications for various *Synechococcus* strains. Clade CRD1a, containing the MITS9220 sequence of interest in this study, is indicated by underlining. The operon structure for the CK_1342 cluster is given (below): single-strand DNA binding protein (yellow), the MreBCD rod-shaped determining complex (pink), and a response regulator (grey) flank the putative sugar-binding protein MsBP (blue). **(b)** A typical architecture of a transporter system defined for maltodextrin^33^ with SBP, transmembrane (TMD), and nucleotide-binding (NBD) domains are indicated. **(c)** operons for predicted interacting partners of MsBP**,** a transmembrane protein (LacF, Cluster CK_1450) and an ATPase (LacG, Cluster CK_1499) are shown with their respective gene numbers and annotated gene products.

For insight into the evolution of putative sugar-binding proteins within sequenced cyanobacterial genomes, 56 orthologous sequences from the Cyanorak cluster CK_1342^14^ were analysed. These cyanobacterial sequences are highly remote from validated examples of sugar-binding SBPs when visualised on the same phylogenetic tree (data not shown). The derived phylogenetic tree depicts five clear branch-points that group according to clade-level classifications (Fig. 1), yet do not correspond to ecological niches or spatio-temporal distributions. This observation is reinforced by gene abundance data from the TARA Oceans dataset^25,26^, highlighting the abundance of the CK_1342 gene across a wide geographical distribution encompassing both coastal and open-ocean environments (Supporting Information Fig. S1).

The CK_1342 cluster is highly conserved across all *Synechococcus* clades, however, it is only present in two extreme low-light *Prochlorococcus* strains *(*MIT9303 and MIT9313*)* and three *Cyanobium* strains. The phyletic conservation of CK_1342 proteins within *Synechococcus* isolates thus alludes to a potentially fundamental role of MsBP in *Synechococcus* metabolism that has been retained under different selection pressures, rather than for specialised niche adaptation in isolated geographical areas. Analysis of the TARA oceans datasets reveals a strong correlation between the abundance of this gene in metagenomes and metatranscriptomes in low-carbon environments, implying its function is predominately related to competitive acquisition of carbon sources across a range of low-carbon environments (Supporting Information Fig. S1).

Cluster CK_1342 proteins are currently predicted to bind chitobiose, a dimer of β-1–4-linked N-acetylglucosamine (GlcNAc), based on similarity to a substrate binding protein, ChiE (KEGG K17244) from the Thermotogae phylum^27^. This ChiE substrate prediction in *T. maritima* is inferred from its presence in an operon containing predicted chitinases (β-*N*-acetyl-glucosoaminidase, chitin deacetylase), and isomerase enzymes under the regulation of a chitobiose ROK-family regulator^27^ but remains experimentally unvalidated. While chitobiose (a GlcNAc dimer) has not been tested as a putative ligand partner of MsBP, we infer that it is unlikely to be the cognate ligand of MsBP, as *Synechococcus* isolates lack an identified pathway for its degradation and shows no detectable affinity for the GlcNAc monomer (compounds tested are presented in Supporting Information Table 1). The metabolic pathway of *T. maritima* ChiE operon terminates at the production of fructose-6-phosphate^28^, which is involved in glycolysis. Therefore, in *Synechococcus*, CK_1342 proteins may mediate the uptake of an intermediary to funnel into this pathway.

The genomic context of MsBP from *Synechococcus* strain MITS9220 indicates an operon structure which is highly conserved among other marine *Synechococcus* strains, stylised in Fig. 1a, and unusual in that it is found as an orphan, not transcribed with the greater ABC-transporter machinery (transmembrane and ATPase subunits) as is typically found in other species (Fig. 1b). Instead, this operon encodes a predicted single-strand DNA-binding protein, the rod-shape determining proteins MreBCD and response regulator SrrA, and more closely resembles that of the MreBCD operon from *E. coli*. From its genomic context, a limited functional inference can be made regarding the MsBP ligand partner and its metabolic role.

ABC transporters have been previously demonstrated to utilise multiple SBPs for the uptake of some nutrients, such as quaternary ammonium compounds for osmoprotection in *Pseudomonas* species^18^, iron uptake^19^, amino acids (ArgT-HisJQMP)^29^, and the oligopeptide uptake system of *Borrelia burgdorferi* which utilises three chromosomally-encoded SBPs and two plasmid-borne SBPs. In the case of the choline-specific ABC transporter (CbcXWV) found in *Pseudomonas syringae* and *P. aeruginosa*, the recruitment of additional carnitine- and betaine-specific SBPs (CaiX and BetX, respectively) allows the translocation of multiple quaternary ammonium compounds with high specificity. The genomic organisation of these additional SBPs is similar to those found in MsBP in that they are also contained in operons without accompanying ABC transporters. The recruitment of such orphan SBPs creates competition for access at the ABC transporter itself, but co-opting existing cellular machinery under stress conditions may ultimately represent a positive trade-off, when compared with the de novo synthesis of all transporter components^30^.

Bioinformatics predictions within the Cyanorak database indicate that the protein encoded by the CK_1342 cluster likely interacts with a putative ABC sugar transporter membrane component (LacF) and permease component (LacG) within clusters CK_1450 and CK_1499, respectively (Fig. 1c). These predictions are based on the strain distribution of these transporter components and a degree of commonality between predicted substrates (lactose). While the *lacF* and *lacG* genes are conserved within the sequenced *Synechococcus* isolates, whether these together result in a competent ABC transporter for sugar uptake in *Synechococcus* isolates remains an open question.

### Ligand binding properties of MsBP and Zn-MsBP

**Figure 2:**
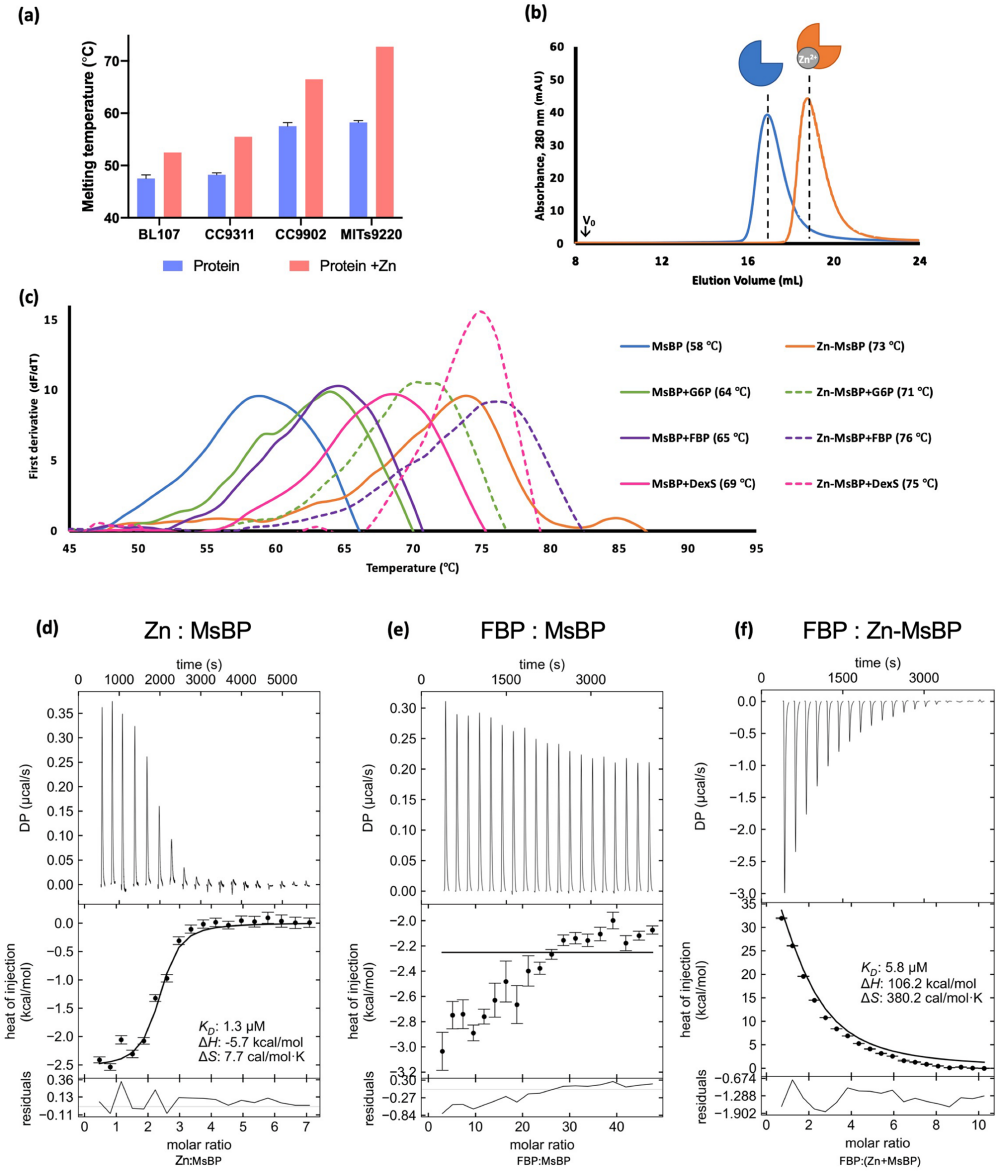
Solution characterisation of MsBP and orthologues. **(a)** Melting temperature of representative CK_1342 proteins in the presence and absence of Zn^2+^ highlighting the additional thermal stability conferred by Zn^2+^. **(b)** Analytical SEC highlighting the observed retention volume of MsBP (blue) and its corresponding derived M_R_ (consistent with a monomeric distribution), which was retarded upon adding Zn^2+^ (Zn-MsBP, orange) seen by the shift in observed retention volumes on a Superdex 200 10/300 GL column made of agarose-dextran composite. **(c)** Ligand screening (fluorescence response) of MsBP was obtained (blue), identifying a high-affinity interaction with Zn^2+^ (Zn-MsBP, orange), and selected sugar derivatives glucose-6-phosphate (G6P, green), fructose-1,6-bisphosphate (FBP, purple), and dextran sulphate digest (DexS, pink). Dashed lines indicate a condition in the presence of Zn^2+^. Curves are presented as the first derivatives of fluorescence response and temperature (dF/dT), where peak maxima correspond to T_*M*_. Analytical SEC procedures utilised an AKTA Pure, operating at 0.5 mL min^-1^ with a Superdex 200 10/300 column. Columns were equilibrated in HEPES buffer (50 mM, pH 7.4), containing NaCl (300 mM) and glycerol (5% v/v). Void volumes for each SEC trace are indicated (V_0_). ITC studies of purified MsBP titrated with **(d)** zinc, **(e)** fructose-1,6-bisphosphate and **(f)** fructose-1,6-bisphosphate after pre-incubation with Zn^2+^ (Zn-MsBP). The heat of injection (kcal/mol) in all cases represents per mol of MsBP.

Orthologous genes from Cluster CK_1342 originating across four different marine *Synechococcus* strains; BL107 (clade Iva), CC9311 (clade I), CC9902 (clade Iva), MITS9220 (clade CRD1a) were purified as a monodisperse product (Supporting Information Fig. S2) and subjected to high-throughput crystallisation trials and determination of melting temperature (T_*M*_, Fig. 2a). As only one, from MITS9220 (referred to as MsBP), produced diffraction-quality material, more detailed solution-state characterisation was undertaken for this individual target.

Solutions of MsBP in HEPES buffer (50 mM, pH 7.4, 300 mM NaCl, 5% v/v glycerol) were established by analytical SEC (shown in Fig. 2b) to constitute a monomer of 43 kDa, with no evidence of higher-order oligomerisation. The thermal melting curve for MsBP was monitored by DSF, as depicted in Fig. 2c, and shows a single transition point (melting temperature, T_*M*_) of 58 °C consistent with a stable, folded entity.

To monitor specific binding preferences of MsBP*,* ~ 400 compounds (HR2-096 Silver Bullets screen; Biolog PM1,2,4a) were mixed with samples of MsBP (μM range) and fluorescence responses recorded across a thermal gradient. Chemical screens encompassed common bacterial metabolic nutrients (C, N, S, P sources), trace metals, and standard protein co-factors. These screens additionally included chemistries (aldose/ketose, amino- and phospho-derivatised sugars, and nucleosides) related to the annotated binding partner of MsBP such as glucosamine sugars (Supporting Information Table S1). As depicted in Fig. 2c, an elevation of MsBP thermal stability (ΔT_*M*_ 6–11 °C) was noted in presence of fructose-1,6-bisphosphate (FBP, T_*M*_ 65 °C), glucose-6-phosphate (G6P, T_*M*_ 64 °C), and a commercially-sourced digest of dextran sulphate (DexS, T_*M*_ 69 °C). However, the MsBP protein did not show any evidence of thermal stability in the presence of unmodified sugars, monosaccharides, disaccharides, and longer (n = 3–10) sugar groupings. Chitobiose, the predicted ligand, was not tested due to difficulties sourcing the required compound.

A significant ΔT_*M*_ of 15 °C was observed for MsBP when mixed with a cocktail of divalent cations (Ca^2+^, Mg^2+^, Mn^2+^, Zn^2+^, data not shown). Following chemical deconvolution, Zn^2+^ was identified as solely responsible for this effect (T_*M*_ 73 °C, Fig. 2a,c). This marked stability caused by Zn^2+^ was also reproduced across orthologues purified from other *Synechococcus* isolates (Fig. 2a). Samples of the native MsBP were cleaved of their N-terminal His-tag and screened, showing a similar DSF shift in ΔT_*M*_ in the presence of Zn^2+^. No DSF response for either His-tagged or cleaved variants was noted for other metal cations (data not shown). DSF screening across our compound library was repeated with Zn^2+^ present in molar excess in the MsBP solution (Zn-MsBP**)**. As shown in Fig. 2c, Zn-MsBP shows higher thermal stability in the presence of FBP (T_*M*_ 76 °C), G6P (T_*M*_ 70 °C), and DexS (T_*M*_ 75 °C), compared to zinc-devoid MsBP in the same conditions.

Detected interactions between dextran digest and MsBP further explains the anomalous solution behaviour on SEC columns composed of cross-linked dextran used in analytical SEC. Following the addition of zinc to MsBP (1:1 stoichiometric ratio) to generate Zn-MsBP, the elution volume (ΔV_e_) was retarded from 16.7 mL (*i.e.*, typical for a ~ 40 kDa species) to 18.9 mL (Fig. 2b). With hindsight from the DSF results, this is now well explained by an altered avidity for the dextran matrix in the presence of zinc.

The specific binding affinity, K_*D*_*,* for interactions between Zn^2+^ and MsBP was measured by ITC and found to be 1.3 + 0.03 µM (Fig. 2d) with a stoichiometry of n = 2.5 (Zn^2+^):1(MsBP**)**. ITC with other transition metals (Mn^2+^, Co^2+^, and Cu^2+^) was also performed, showing either no (Mn^2+^) or negligible (> 1 order of magnitude lower, Cu^2+^, Co^2+^) interaction, verifying the specificity of the observed Zn^2+^ interaction (data not shown).

To determine if zinc cooperatively promotes substrate recognition by MsBP, we investigated the interaction of MsBP and Zn-MsBP with putative ligand candidates (FBP and G6P) with ITC. While ITC data showed weak interaction of zinc-devoid MsBP for FBP (Fig. 2e), and no interaction for G6P (data not shown), pre-incubation of the protein with Zn^2+^ to generate Zn-MsBP (10 × molar excess) detected a strong endothermic interaction, specifically between Zn-MsBP and the disubstituted FBP, K_*D*_ = 5.8 µM (Fig. 2f). No measurable interaction of Zn-MsBP was observed for the monosubstituted phosphate derivative, G6P, or the control condition, maltose (data not shown). Our ITC data thus indicates a strong requirement for Zn^2+^ to bind sugar compounds and preference of MsBP for sugars embellished with multiple anionic substituents.

We note all *Synechococcus* species within Cyanorak are annotated to possess class II fructose-1,6-biphosphatases (Cyanorak cluster CK_878) with low similarity to conventional FBPases^31^, providing a possible mechanism for downstream processing of such a substrate source. In considering phosphate-embellished sugars, it is essential to note that this does not exclude the potential for sulphated polysaccharides, such as fucoidan and carrageenan (standard components of Rhodophyta (red algal) cell wall and extracellular matrix) to form the cognate ligand of this SBP, however systematic testing of such compounds is currently unfeasible due to difficulty sourcing them. Under typical conditions, such sulphated polysaccharides act as ionic and osmotic regulators and could contribute to labile marine dissolved organic material (DOM) during turnover due to predation. Likewise, marine cyanobacteria (*Synechococcus spp.* WH8102 and CC9311) have been shown to incorporate sulphates into membrane lipids and replace phosphate in LPS with other negatively-charged moieties (such as galacturonic acid)^32^, representing other sources of negatively charged substrates present within the labile DOM pool that MsBP may recognise.

### Crystal structures of MsBP in Zn-free and Zn-bound form

**Figure 3:**
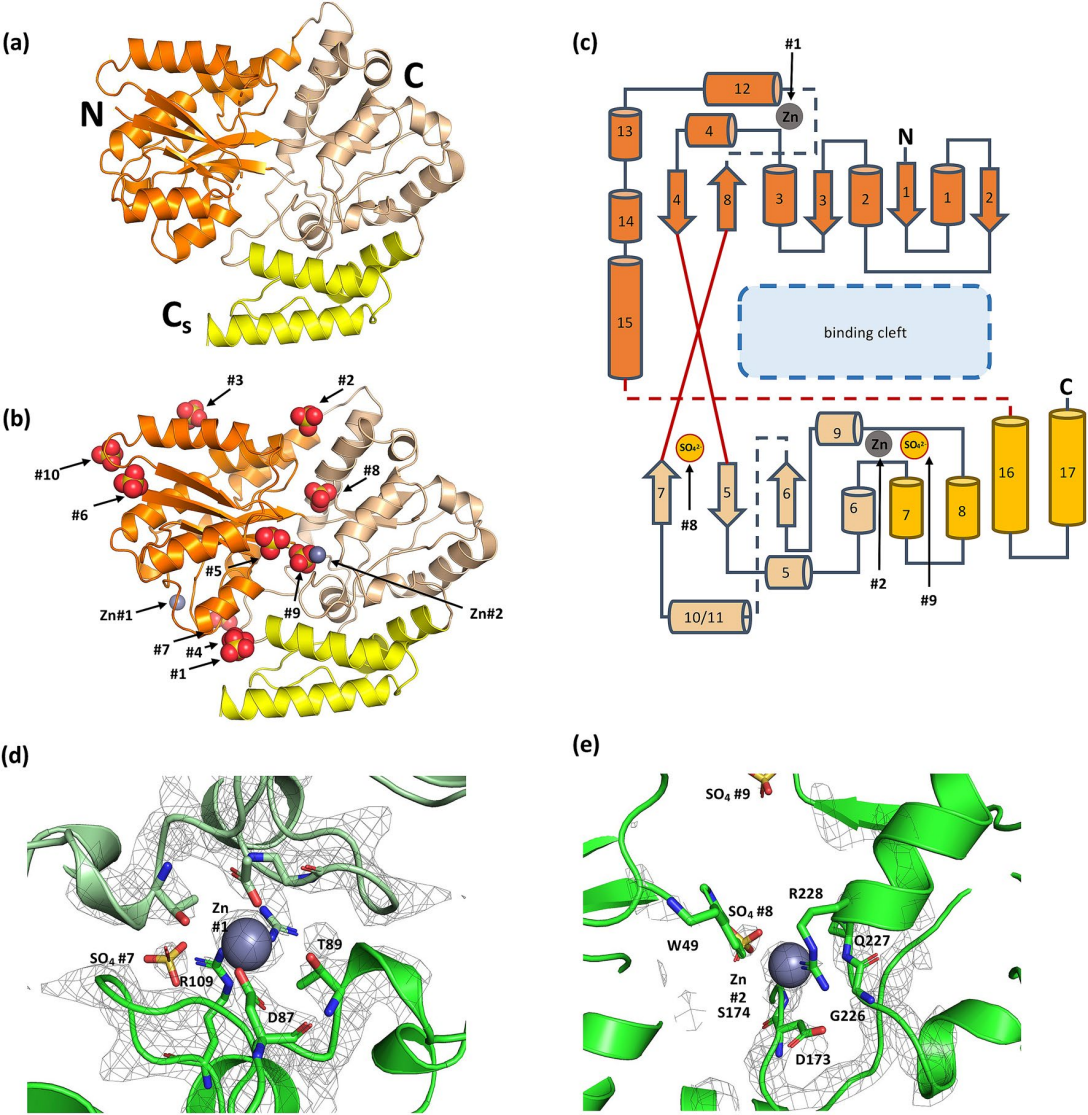
Crystal structure of MsBP and Zn-MsBP. **(a)** The cartoon representation of the MsBP structure showing the N-terminal domain (orange), C-terminal domain (wheat), and a C-terminal subdomain (yellow) characteristic of structural cluster D-I. **(b)** Structure of Zn-MsBP with identical domain colouring includes bound sulphate (red) and zinc ions (grey). **(c)** The topology map for MsBP (and Zn-MsBP), highlighting the approximate location of the bound zinc sites and sulphate ions found in the binding cavity. Dashed lines indicate elements passing behind the plane. The substrate-binding cleft is shown (blue box). **(d)** The crystal structure of Zn-MsBP (6WPM) is shown with electron density overlaid at Zn site 1 and **(e)** Zn site 2. Electron density is shown as 2F_0_-F_C_ map contoured at 2σ. For Zn site 1, the monomer within the unit cell is shown as dark green, with the neighbouring monomer from the adjacent unit cell shown in light green contributing identical co-ordinating residues.

Crystal structures (Fig. 3) for MsBP (PDB: 6WPN) and Zn-MsBP (PDB: 6WPM) were determined to a resolution of 2.3 Å and 2.8 Å, respectively. The structure of Zn-MsBP showed a single chain containing two Zn^2+^ atoms in the asymmetric unit (a.s.u). The structure of MsBP displayed two chains in the a.s.u. No electron density attributable to a ligand was distinguished in either structure, indicating both proteins were crystallised in ligand-free forms. Notably, the structure of Zn-MsBP shows ten molecules of SO_4_^2−^, possibly sequestered from the sulphate rich crystallisation condition (2 M (NH_4_)_2_SO_4_, 20% w/v PEG3350). The metal-free MsBP also crystallised in a sulphate rich condition (2 M (NH_4_)_2_SO_4_, 0.1 M Tris, pH 8.5), however, does not show any electron density attributable to a SO_4_^2−^ ion.

As outlined in Fig. 3, the MsBP and Zn-MsBP structures adopt a classical SBP fold of two α/β domains oriented around a binding cleft and connected by a hinge region of two short β-strands. A third crossover is noted between a distinct helical sub-domain (a hairpin of helices α16 and α17) which makes close contact with the C-terminal domain. The core β-sheet of the N-terminal domain consists of 5 strands in topology β_2_β_1_β_3_β_8_β_4._, whereas the C-terminal sheet comprises three antiparallel strands. The intervening binding cleft shows a markedly positive charge, suggestive for orienting a negatively charged substrate. The β-sheet topology and the number of domain crossovers are consistent with the designation of MsBP within Cluster D of the SBP superfamily^21,22^. The presence of the additional C-terminal subdomain places it unambiguously within sub-cluster D-I, associated with a narrow substrate preference for carbohydrates^22^.

In the Zn-MsBP crystal structure, additional electron density consistent with a heavy atom is noted at two sites. We attribute both as zinc atoms, consistent with zinc edge absorption observed during data collection. This occupancy is broadly consistent with stoichiometry (*n* = 2.5) determined for Zn-binding by ITC (Fig. 2). One Zn^2+^ atom (Zn #1) appears at the periphery, weakly held within a negatively-charged cavity of the crystal lattice, created by the side chains of amino acids, such as D87 and T89, from two neighbouring monomers (Fig. 3d). It is unclear if Zn^2+^ at this peripheral location corresponds to a functional site, as seen in the related MBP which uses a structurally similar site to dock to its cognate receptor^33^, or instead reflects a crystallisation artefact forming a contact between adjacent monomers.

The second Zn^2+^ atom (Zn #2) is located within the binding pocket of the protein. At the available resolution (2.8 Å), it is unclear precisely which residues co-ordinate Zn. However, candidate sidechains in the vicinity include S174, Q227 and R228 (Fig. 3e). The fourth coordination for this zinc may be satisfied with the carboxylate group of D173. This zinc is held peripheral to the binding cleft of the protein and is within 4 Å of the aromatic sidechain of W49, positioned over the binding cleft.

**Figure 4:**
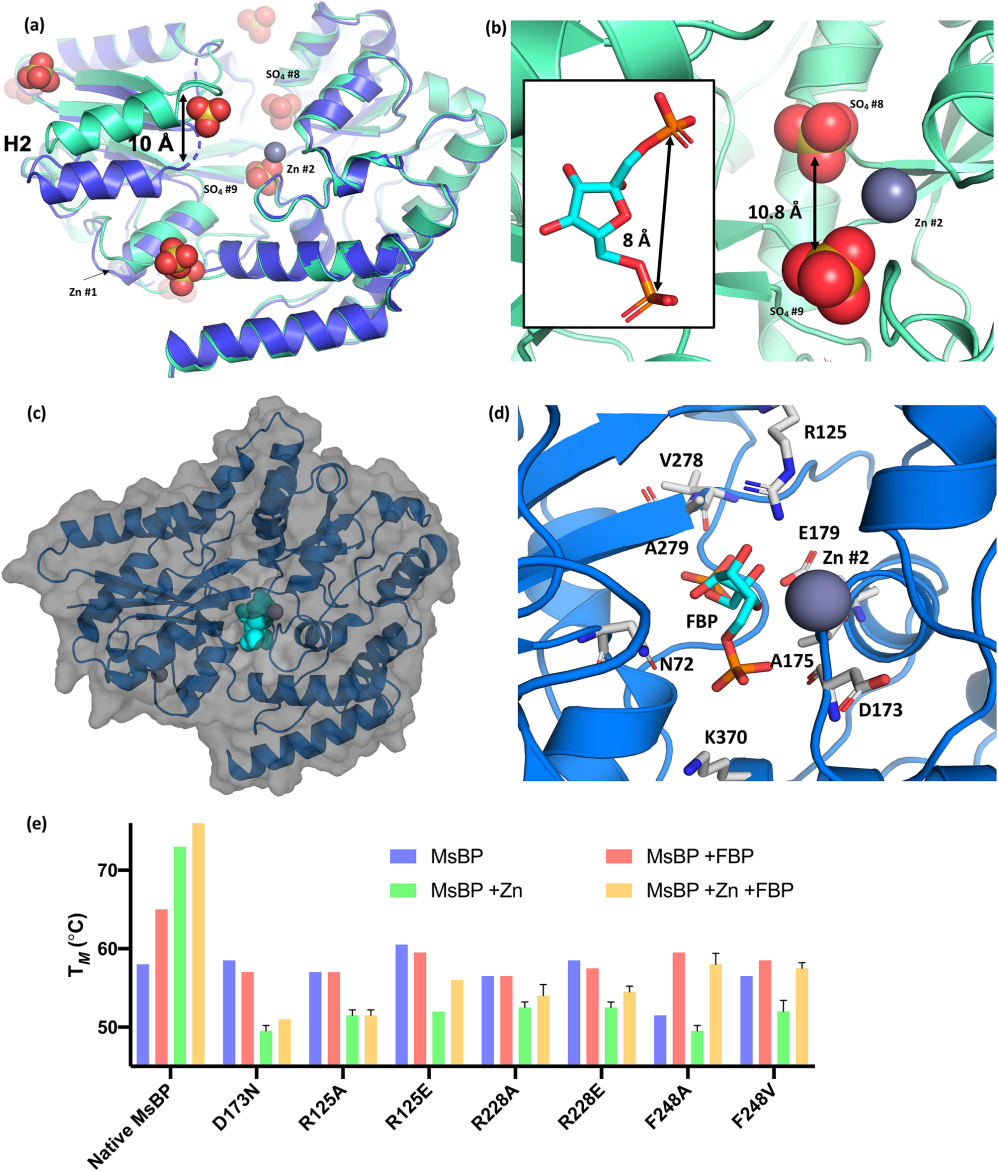
The ligand binding cleft of MsBP. **(a)** depicts an overlay of Zn-MsBP (aqua) and unbound MsBP (blue, r.m.s.d. 0.873 Å) highlighting structural difference caused by movement of helix 2 (10 Å) in the presence of zinc. Key Zn (grey) and sulphate (red) ions are indicated. **(b)** highlights the modelled distance between key sulphate ions captured within the binding cleft of Zn-MsBP with (inset) the distance between cognate sulphate ions for fructose-1,6-bisphosphate (PDB ligand code FBP). **(c)** Best FBP conformer docked in the binding cavity of Zn-MsBP using AutoDock Vina^66^. **(d)** Zoomed view of FBP conformer with best fit (autodock ligand fit number 1) localised at SO_4_^2−^ #9 site within the Zn-MsBP binding cleft, showing predicted phosphate positioning with putative binding residues indicated. **(e)** Thermal melt analysis of MsBP mutants. MsBP melting temperature (violet), as well as in the presence of FBP (red), Zn (green), or FBP + Zn (yellow), are indicated for native MsBP protein and single-site mutants that correspond to the predicted binding residues identified from the docked FBP substrate.

The crystal structure of Zn-MsBP also shows ten sulphate anions, mainly at surface regions of positive charge, as expected. Two sulphate ions are positioned in the positively-charged binding cleft: one adjacent to the bound Zn^2+^ (SO_4_^2−^ #9), and the second buried deeper within the cleft (SO_4_^2−^ #8) (Figs. 3 and 4). We note that the spacing between these captured SO_4_^2−^ ions (SO_4_^2−^ #8 and SO_4_^2−^ #9) may mimic the orientation of adducts of a biphosphate/bisulphate saccharide substrate such as FBP (Fig. 4b), thereby substantiating our ITC data.

### Insights into the binding mode of MsBP to a modified sugar

Overall, the structures of MsBP and Zn-MsBP overlay with r.m.s.d. 0.87 Å (Cα atoms), with the most striking difference caused by marked and localised tilting of helix 2, attributable to a lower degree of conformational variability in loop 3 (Fig. 4a). In Zn-MsBP, helix 2 is pushed ~ 10 Å over the binding cleft, attenuating access to the substrate-binding channel. Dynamic domain analysis^34^ shows a rotation angle of 6.8° and binding cleft closure of 22.2% between the ‘open’ apo MsBP and ‘partially-closed’ Zn-MsBP structural conformations. The observed partial closure of the binding pocket for Zn-MsBP, caused by the movement of helix 2 exposes a hydrophobic patch to solvent, and positions W49 to occlude the entrance to the cleft, closely positioned to the bound Zn^2+^. Thus, from the ‘partially-closed’ conformation of the Zn-MsBP structure**,** it is inferred Zn^2+^ may modulate ligand binding, consistent with an induced-fit mechanism^35^. A comparable analysis of substrate-bound forms of other sugar-binding SBPs (e.g. GacH: α-acarbose-binding protein, *Streptomyces glaucescens*^36^; SO-BP: short oligosaccharide-binding protein, *Listeria innocua*^37^; and TmMBP: maltose-binding protein, *Thermotoga maritima*^38^) show a much larger rotation angle (between 29° and 56°), and complete closure of the binding cleft (> 97%), upon sequestering a bound ligand (Supporting Information Table S2).

Molecular docking of FBP within the ‘partially-closed’ structure of Zn-MsBP (Fig. 4c,d) shows potential engagement of a phosphate-embellished sugar, which aligns well with the positioning of SO_4_^2−^ #9 sequestered within the interdomain cleft of the Zn-MsBP structure. The binding energies for the nine FBP conformers determined are comparable (Supporting Information Table S3). Eight of these conformers overlap spatially at the position of SO_4_^2−^ #9, however, differ with respect to the orientation of phosphate substituents. The ninth conformer localised at the SO_4_^2−^ #8 site.

Examining the proposed binding cleft and various FBP conformers predicted from the ligand docking, potential co-ordinating residues for FBP were identified (Fig. 4d). These include charged residues R125 and D173 as well as the main-chain N-atoms R228 and F248 that appear to stabilise the ligand at the SO_4_^2−^ #8 site (not shown). From the ligand docking, it seems that FBP is not large enough to engage both SO_4_^2−^ sites simultaneously in the ‘partially-closed’ Zn-MsBP structure. While the phosphate embellished sugar clearly engages residues within the binding cleft, these results possibly indicate the cognate substrate of MsBP is either larger than FBP or one of the SO_4_^2−^ sites serves as a channel to direct the substrate deeper within the binding cleft when the protein is in the ‘closed’ ligand-bound state.

### All MsBP binding site mutants show complete loss of sugar engagement

Given the low resolution of the Zn-MsBP crystal structure, further exploration of the modified sugar binding mechanism was performed using site-directed mutagenesis of residues around the proposed binding cavity. These MsBP mutants, R125A, R125E, D173N, R228A, R228E, F248A, F248V, were designed to either disrupt (i) charge, (ii) polarity, or (iii) steric bulk of the residues that may form part of the proposed ligand-binding site. The purpose of this mutagenesis study was to identify if disrupting specific sidechains led to any abrogation of the previously observed thermal stability or behaviour on cross-linked dextran columns, suggesting an essential mechanistic role.

Thermal melt analysis of the single-site mutants was carried out in the presence of zinc, FBP, or a combination of both molecules (Fig. 4e). This analysis highlighted that all MsBP mutants showed decreased thermal stability in the presence of FBP (58 ± 3 °C), in contrast to the increased thermal stability previously observed for the native MsBP (Fig. 2c). Furthermore, adding Zn^2+^ did not lead to higher thermal stability but instead destabilised all MsBP mutants. Incorporating both FBP and zinc in these thermal melt assays marginally stabilised the MsBP mutants, but this was substantially lower than previously observed for the native MsBP.

Native MsBP also showed unique solution-state behaviour in the presence of zinc (Fig. 2b). While all seven MsBP mutants in zinc-free conditions eluted at a volume consistent with a monomeric form (43 kDa), none of the MsBP mutants in the zinc-treated conditions showed the retardation previously observed for wild-type MsBP on the dextran column (Supporting Information Fig. S3).

As single amino acid substitutions of all MsBP mutants appear sufficient to significantly lower the thermal stability and completely abrogate dextran column interactions, functionally R125, D173, R228 and F248 may be interconnected and fulfil an essential mechanistic role. However, the impact of specific sidechains on the engagement of a potential sulphate- or phosphate-embellished ligand are difficult to disentangle, as many protein-anion interactions appear to be mediated by main-chain -NH groups, and the structural resolution of Zn-MsBP is too low to resolve atomic-level details that might otherwise accurately indicate ligand coordination.

### MsBP engages unique binding chemistry compared to other sugar binding proteins

Structural relatives of MsBP and Zn-MsBP were identified by a DALI search demonstrating homology to other sugar-binding proteins of cluster D classified earlier (Table 1). The structural relatives for MsBP and Zn-MsBP were mainly found in the closed-liganded state and display r.m.s.d values > 2.5 Å. This is especially pronounced for Zn-MsBP as a consequence of the movement of helix 2. While the overall fold of the structural homologues are similar, the r.m.s.d value is high. However, this is not inconsistent with other non-classical maltose-binding proteins^39^.

**Table 1:** Structural relatives of MsBP.

PDB	Protein name	Organism	Ligand	Seq. ID (%)	r.m.s.d. /Å		Z-score	
					Zn-MsBP 6WPM	MsBP 6WPN	Zn-MsBP 6WPM	MsBP 6WPN
3K00	GacH	*Streptomyces glaucescens*	Maltotetraose	21	3.4	3.1	35.0	36.5
4QSD	–	*Agrobacterium fabrum*	Sucrose	21	3.6	3.1	34.4	35.1
5CI5	–	*Pseudothermotoga lettingae*	α-d-tagatose	21	3.4	3.1	37.0	37.4
5YSE	SO-BP	*Listeria innocua*	Sophorotetraose	20	3.1	2.7	34.7	36.1
6DTQ	TmMBP	*Thermotoga maritima*	Maltose	21	3.7	3.2	34.7	36.1
6R1B	UgpB	*Mycobacterium tuberculosis*	Glycero-phosphocholine	19	3.2	2.7	34.4	35.4
7BVT	GMMBP	*Arhrobacter globiformis*	Cyclic α-maltosyl-(1→6)-maltose	20	3.5	3.3	35.4	36.0
7C6J	βGlyBP	*Thermus thermophilus*	Cellobiose	21	3.0	2.7	34.6	36.0

**Figure 5:**
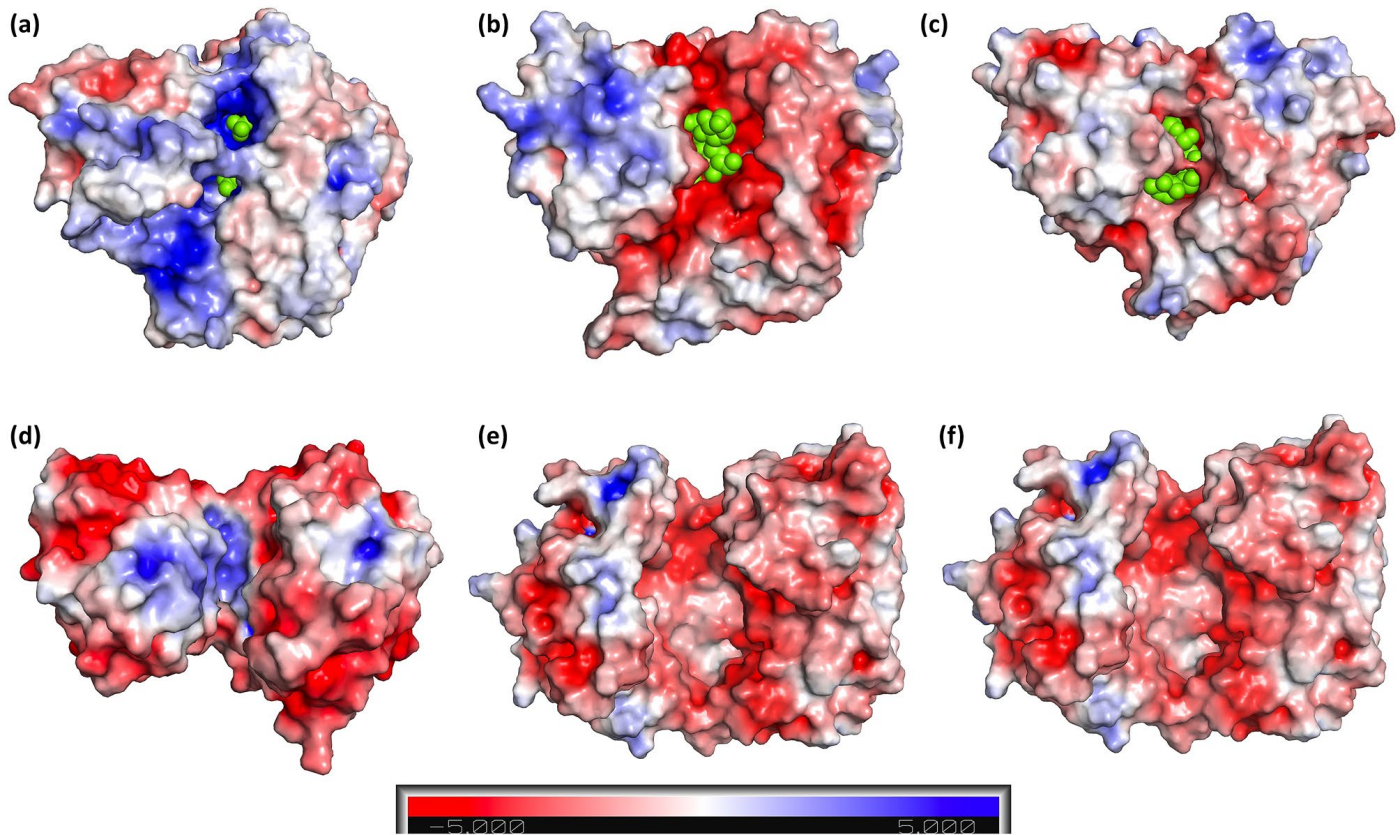
Comparison of binding cleft chemistries for MsBP relatives. The APBS electrostatic surface representation^68^ of Zn-MsBP (6WPM) is shown in **(a)** highlighting the unique positively-charged binding cleft**.** Structurally related sugar-binding proteins **(b)** SO-BP (PDB ID: 5YSE)^37^, **(c)** GacH (PDB ID: 3K00)^36^, **(d)** anion-binding protein ModA (PDB ID: 3K6V)^41^, **(e)** metal-mediated fructooligosaccharide-binding protein(FusA) from *S. pneumoniae* (PDB ID 5G5Y)^69^, and **(f)** the chitin-binding protein (CBP) from marine *Vibrio* sp. (PDB ID 5YQW)^70^ are presented for comparison. All proteins are shown looking directly on the binding cleft with charge coloured at the same scale. Ligands **(b–c)** and bound sulphate ions (Zn-MsBP) are shown as green spheres. Blue corresponds to a positive surface charge; red corresponds to a negative.

Cluster D SBPs cover a diverse array of ligand chemistries (carbohydrates, polyamines, oxyanions, and metal ions)^22^. In cases where ligand screening has failed to yield a candidate for SBPs, the function of orphan SBPs can be inferred by interrogation of binding-site chemistry^40^. As previously discussed, the general nature of the binding cleft of MsBP is positively charged (Fig. 5a), which is markedly different from structural relatives that display negatively charged binding cavity (Fig. 5b,c), appropriate for interaction with simple sugars. As outlined in Fig. 5d, the MsBP binding region is also more strongly positive than oxyanion-binding proteins (MoO_4_^2–^binding ModA)^41^. We ascribe the unusual positive charge seen within the MsBP binding cleft to the need to sequester sugar adducts with phosphate or sulphate embellishments, likely originating from sources such as degradation products of micro- and macroalgal extracellular glycans^42^ or cyanobacterial lipopolysaccharide^32^.

**Figure 6:**
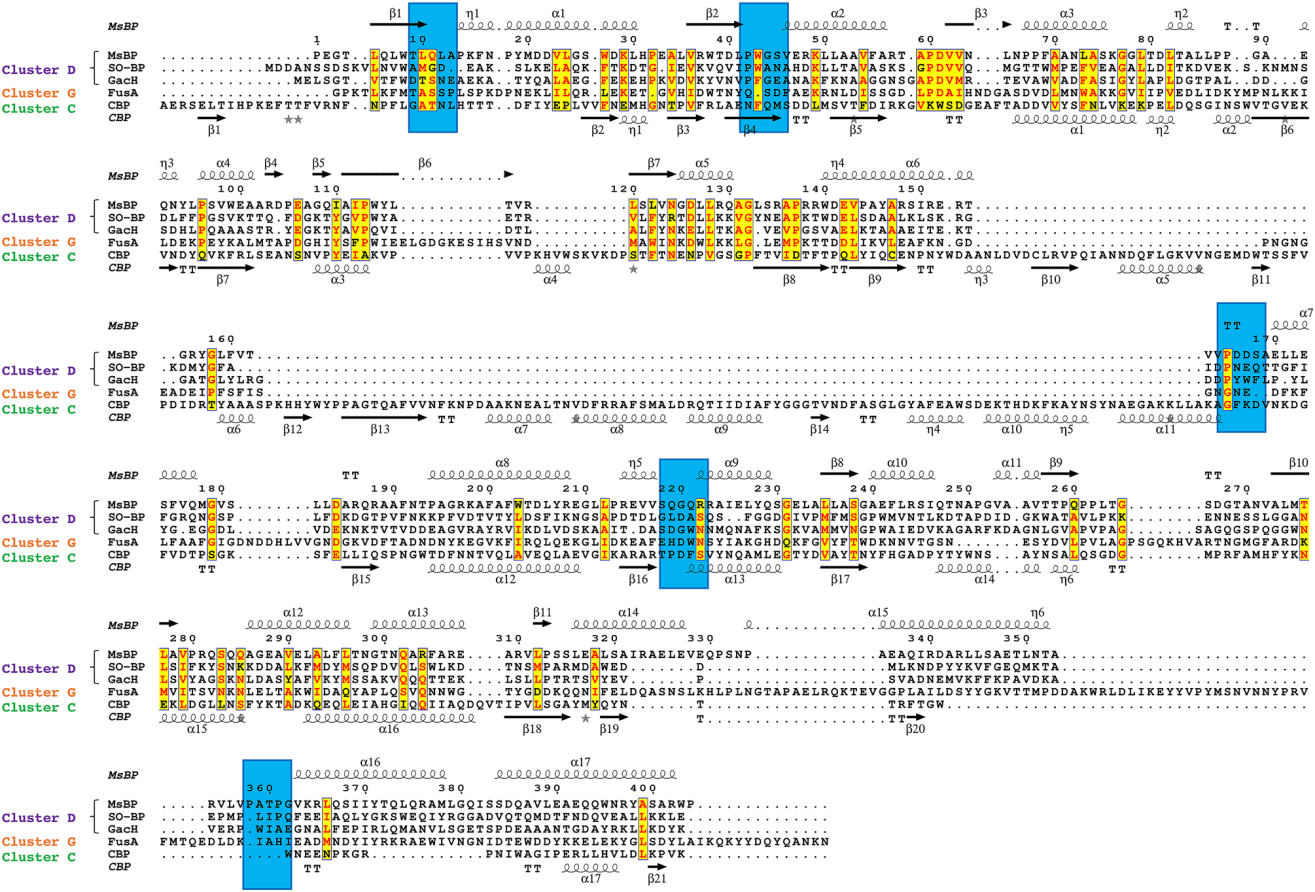
Structural based sequence alignment of MsBP relatives*.* Sequences of cluster D proteins; SO-BP (PDB ID: 5YSE)^37^ and GacH (PDB ID: 3K00); cluster G protein FusA (PDB ID 5G5Y)^69^ and cluster C protein CBP (PDB ID 5YQW) are aligned with MsBP sequence. Conserved secondary structure elements forming ligand recognition sites where applicable are highlighted (blue box). Residues with conserved chemistry are shown in yellow. Figure generated with ESPript^71^.

Comparisons to other oligosaccharide-binding proteins in structural clusters C and G^21^ also highlight the unique nature of the MsBP binding cleft. Structure-based sequence alignment of representative sugar-binding relatives from SBP cluster C, D and G is presented in Fig. 6, highlighting similarity and differences between the secondary structure elements. The Ca^2+^-mediated fructooligosaccharide-binding protein (FusA; cluster G) from *S. pneumoniae* shows a more spacious, negatively-charged binding cavity to accommodate a bulky oligosaccharide ligand (Fig. 5e). In this protein, the co-ordinated Ca^2+^ ion is also found away from the binding cleft in a conserved EF-hand motif. The chitin-binding protein (CBP; cluster C) from marine *Vibrio* sp. (Fig. 5f) also features a negatively-charged binding cleft and originates from an entirely different structural grouping that is markedly different from MsBP. It is unknown if the chitin-binding protein also binds chitobiose, however, the markedly different structural arrangement and binding cleft chemistry support our inference that MsBP is unlikely to bind chitin or, by extension, the related chitobiose as predicted.

## Conclusion

Bioinformatics analyses indicate marine picocyanobacteria apportion large parts of their transport capacity to proteins predicted to facilitate survival in nutrient-poor environments. Our ligand screening of MsBP from *Synechococcus* MITS9220 highlights a zinc-mediated preference for phosphate (or sulphate) embellished sugar, fructose-1,6-bisphosphate. Such sugars could be released from extracellular glycans in micro- and macroalgae^42^ or from cyanobacterial lipopolysaccharide^32^ and represent an underappreciated portion of the accessible microbial nutrient pool within the ocean^43^. These results correlate with the highly positively charged binding cavity in the crystal structures of MsBP.

The unique substrate preference of MsBP likely represents a highly specialised function. The sequence alignments generated for phylogenetic analysis highlight many of the key proposed binding residues are conserved across all major *Synechococcus* clades. We postulate MsBP plays a fundamental biochemical role rather than being a consequence of niche adaptation. The observed interaction of modified sugars with MsBP lends further evidence to the proposed uptake of sugars by *Synechococcus*, possibly as a mixotrophic strategy. Our MsBP characterisation also highlights that annotations arising from remote homology, while representing a ‘best guess’ to function, fail to capture the diverse repertoire of nutrient sources available in marine environments as ligand partners, obscuring accurate functional assignments. Future work in this area could aim to elucidate the precise, underlying mechanisms for zinc-mediated substrate tuneability and supplementing the available in vitro evidence with robust physiological studies to identify the overarching elements and function of the metabolic pathways in which MsBP is involved.

## Experimental procedures

### Genomic and phylogenetic analyses

The Cyanorak database (https://www.sb-roscoff.fr/cyanorak) is a repository for 97 sequenced cyanobacterial genomes (48 unique *Synechococcus*, 43 *Prochlorococcus,* and 6 *Cyanobium*)^14,44^. One gene cluster in this database, CK_1342, contains 56 orthologous sequences and is annotated as a putative sugar-binding protein, based on the similarity to a KEGG cluster (K17244) predicted to bind chitobiose^27^. Phylogenetic analysis^45^ was performed using 56 unique CK_1342 sequences (average sequence ID = 70%). Following a preliminary multiple sequence alignment, computed using the L-iNS-I option of MAFFT^46^. The phylogenetic tree was inferred using IQ-Tree^47^, with the optimal model, using the TESTONLY option, found to be LG + F + I + G4. The inferred maximum likelihood model was used to generate a final phylogenetic tree, visualised using iTOL^48^.

### MsBP construct design

The gene *MITS9220_00117*, annotated to encode a putative sugar-binding protein (MsBP, *m*odified *s*ugar-*b*inding *p*rotein) from the marine *Synechococcus* sp. MITS9220 was selected for detailed characterisation. Other targets referred to in this investigation include *BL107_06034*, *CC99092_00152*, and *CC9311_00114*. Gene sequences for these targets are available from the Cyanorak v2.1 database (https://www.sb-roscoff.fr/cyanorak). Sequences were analysed using the SignalP4.0 server^49^, which predicted the presence of a twin-arginine (Tat) signal peptide. To facilitate purification, this was removed, and the required gene PCR-amplified from genomic DNA (extracted using the CTAB/phenol–chloroform method), incorporating vector-specific pET15-b overhang regions*.* Ligation-independent cloning^50^ into the pET15-b vector was carried out using *BamHI* and *NdeI* restriction sites to incorporate an N-terminal hexahistidine tag with a thrombin cleavage site. Transformants were verified by sequencing.

### Protein expression and purification

Following transformation, *E. coli* DE3 BL21 cells were grown (500 mL cultures in 2 L baffled flasks) at 25 °C, 200 rpm for 24 h using the autoinduction method^51^. Heavy atom protein derivatives were prepared for experimental phasing in crystallography experiments, using commercially sourced growth media defined for selenomethionine (SeMet) incorporation (Medcilon). For SeMet derivatives, growth was monitored until OD_600_ reading 1.2 (SeMet) was achieved, recombinant expression was induced with isopropyl β-D-thiogalactopyranoside (final concentration 1 mM), and cells grown overnight.

For all preparations, cells were harvested by centrifugation (4000*g*), resuspended in HEPES buffer (50 mM, pH 7.4) with imidazole (5 mM) and NaCl (300 mM), and lysed with a single freeze–thaw step (liquid N_2_) and sonication on ice. Clarified lysate (30 mL) was loaded with a peristaltic pump onto a prepacked Ni–NTA column (1 mL) pre-equilibrated in the same buffer. The protein product was eluted by addition of 5 column volumes of imidazole (500 mM) in HEPES buffer. Eluate fractions were pooled and desalted using size-exclusion chromatography (SEC) (Superdex HiLoad 200 16/600 column, GE Healthcare) in HEPES buffer (50 mM, pH 7.4) with NaCl (300 mM). Reducing agent tris(2-carboxyethyl)phosphine (TCEP, 0.5 mM) and glycerol (5% v/v) were included in all protein purification buffers. SeMet preparations additionally contained EDTA (1 mM).

Protein-containing fractions were concentrated using centrifugation (10 kDa MWCO), and snap-frozen in liquid N_2_. The purity of the recovered His_6_-tagged product was verified using SDS-PAGE, showing a single band at 45 kDa.

To remove the affinity tag, purified native protein (4 mg mL^−1^) was treated with thrombin (40 U mL^−1^) for 8 h at 32 ºC, before the cleaved tag and thrombin were removed (GE HiTrap NiNTA and Benzamidine, connected in tandem). Protein flow-through was spin-concentrated and cleavage verified by SDS-PAGE and anti His Western blot (ThermoFisher).

### Analytical size-exclusion chromatography

The evaluation of native protein mass in solution was carried out using analytical SEC on a Superdex 200 10/300 GL column (GE-Healthcare) equilibrated in HEPES buffer (50 mM, pH 7.4) with NaCl (300 mM) and glycerol (5% v/v). Calibration of elution times was carried out using commercial size standards (GE-Healthcare), and void volume (V_o_) estimated using blue dextran. Partition coefficients (K_av_) were calculated from elution volumes and used to derive a plot of K_av_ against log(M_R_) to allow the interpolation of unknown masses based on elution volume. The line of best fit was given as *log(M*_*R*_*)* = *-3.748(K*_*av*_*)* + *6.593*, with a correlation coefficient (R^2^) of 0.9957. Notably, prior to the analytical SEC, all MsBP mutants were dialysed overnight in HEPES buffer containing 1 mM EDTA to strip any co-purified metal ions.

### Differential scanning fluorimetry (DSF)

Commercially-available libraries of small molecules (HR2-096 Silver Bullets, Hampton Research and MicroPlates PM1–PM4, Biolog Inc.) in millimolar concentrations (1–10 mM) were tested as potential binding partners to native MsBP by recording DSF responses over a thermal gradient^52^. SYPRO Orange dye (Invitrogen) was used to monitor fold integrity in protein mixtures (protein concentration 10–50 μM) by measuring fluorescence at 590 nm following excitation at 485 nm using a Stratagene MX3000P RT-qPCR instrument. Each condition was tested in triplicate, with each 96-well plate containing a control well with no additive.

The raw curves were analysed using the analysis template provided^52^ and fitting of Boltzmann distribution (GraphPad Prism) for the determination of mid-point thermal melt temperatures^53^. Conditions leading to a significant increase (> 3 °C) in melting temperature were deconvoluted to identify the single component leading to such a change and repeated in triplicate.

To exclude the effects of metal ion-buffer interactions, purified MsBP was thawed, dialysed for two hours into MES buffer (50 mM, pH 5.6) with NaCl (300 mM), EDTA (1 mM), and glycerol (5% v/v), and then overnight into the same buffer without EDTA.

### Isothermal titration calorimetry (ITC)

MsBP samples were dialysed overnight into HEPES buffer (50 mM, pH 7.4) with NaCl (300 mM) and glycerol (5% v/v) before being concentrated to 50 μM or greater. Ligands (analytical grade, SigmaAldrich) were prepared as stock solutions (0.1 M) in acid-washed volumetric flasks and polyethylene equipment to prevent trace metal contamination. To minimise artefacts due to buffer mismatches, overnight dialysis buffer was filtered, degassed, and used to prepare ligand stocks and working solutions.

Binding interactions were analysed using a Nano-ITC (TA Instruments) with stirring at 300 rpm in a cell maintained at 25 °C, and a medium power setting. Following a priming injection (1 μL for 1 s), 19 aliquots (2.5 μL) were injected, each at 4 s duration spaced at 300 s, with a filter period of 5 s. Initial titrations used a tenfold excess ligand, with ligand-to-protein ratios optimised following inspection of initial thermograms. For each analysis, a buffer/salt solution without protein was used as a blank to ascertain the contribution of the heat of dilution. Raw files were exported to Nitpic^54^ for baseline detection, subtraction of blank, and peak integration, before isotherms were plotted with Sedphat^55^ and visualised using GUSSI (http://biophysics.swmed.edu/MBR/software.html).

### Crystallisation, data collection, and structure determination

Aliquots of SeMet-derivatised MsBP (12 mg ml^−1^) in HEPES buffer (50 mM, pH 7.4), NaCl (300 mM), glycerol (5% v/v) were subjected to ten distinct sparse-matrix screens from three commercial sources (Hampton Research, Molecular Dimensions, and Rigaku). MRC-2 well UVP plates (Swissci, Switzerland) in a 96-well format were set up at the Structural Biology Facility—Mark Wainwright Analytical Centre (UNSW) using a Phoenix robot to dispense 200 nL sitting drops containing 1:1 protein-to-precipitant. All screens were repeated with the addition of zinc chloride (4 mM). Trays were stored at 22 ºC, with crystals appearing after 1–2 months.

Diffraction-quality crystals of apo MsBP were observed in the following condition: (NH_4_)_2_SO_4_ (2 M) and Tris (0.1 M, pH 8.5). The addition of zinc resulted in diffraction-quality crystals in (NH_4_)_2_SO_4_ (2 M) and PEG3350 (20% w/v). These crystals were harvested using a nylon loop, cryoprotected by soaking briefly in mother liquor with the addition of 20% PEG200, and flash-frozen in liquid N_2_. While optimisation of crystallisation conditions was trialled, it did not improve the quality of diffraction. Several attempts were made to co-crystallise Zn-MsBP with FBP, including substantial seeding experiments, however, this proved unsuccessful.

Data collection was carried out remotely using the MX2 beamline at the Australian Synchrotron (Melbourne, Australia). Reflections were measured on an Eiger X 16 M detector (Dectris, Switzerland) and data collected using Blu-Ice software^56^. For Zn-MsBP selenomethionine-derivatised crystals, single-wavelength anomalous diffraction (SAD) data was collected at a wavelength of 0.9791 Å (12.66 keV) and 10% attenuation to allow experimental phasing^57^. The presence of zinc within the crystal lattice of Zn-MsBP was confirmed by a strong absorbance peak at the zinc edge (~ 9.6 keV). For the metal-free MsBP, data were collected at a wavelength of 0.9537 Å (12.99 keV) with a 10% attenuation. Diffraction data for Zn-MsBP crystals were indexed, integrated, and scaled using the XDS suite^58^. For the native crystal dataset, these steps were performed using DIALS^59^.

The Zn-MsBP structure was solved by experimental phasing using Se-SAD^57^. Diffraction was observed to 2.23 Å but was trimmed to a resolution of 2.8 Å in space group P4322 due to extensive radiation damage. A partial model was generated using PhaserEP in Phenix^60^ from 3 Se atom sites. Due to low initial completeness (~ 25%, 7 partial chains), extensive manual building in *Coot*^61^, and iterative refinement in PHENIX.REFINE was required. This we attribute to intrinsically high B factors (average 90 Å^2^). The final coordinates contain residues 10–410 in one chain, with 2 defined zinc sites and 10 sulphate anions. The final structure contains a surprisingly low solvent content, with only 6 ordered water molecules distinguishable from the bulk solvent, a R_free_ value of 0.25, and 2.5% of sidechains in outlier conformations. Final refinement statistics are given in Table 2, and coordinates are deposited in the PDB (6WPM).

**Table 2:** X-ray data collection and refinement statistics.

Structure PDB code	Zn-MsBP 6WPM	MsBP 6WPN
**Data collection**^**a**^		
Wavelength (Å)	0.9792	0.9537
Space group	P 43 2 2	P 1 21 1
Molecule/asymmetric unit	1	2
**Unit-cell dimensions**		
a, b, c (Å)	144.78, 144.78, 53.72	59.95, 69.53, 83.72
α, β, γ (°)	90.00, 90.00, 90.00	90.00, 94.70, 90.00
Resolution range (Å)	48.26–2.88 (3.04–2.88)	46.79–2.29 (2.37–2.29)
*R*_merge_ (%)	3.2 (2.3)	7.2 (2.8)
I/sig(I)	12.7 (27.2)	9.5 (1.5)
Completeness (%)	99.9 (99.5)	99.0 (90.7)
Anomalous completeness (%)	99.8 (98.6)	–
Multiplicity	1.8 (1.9)	4.9 (1.0)
Anomalous multiplicity	1.0 (1.0)	–
**Refinement**^**b**^		
Resolution range (Å)	48.2–2.88	46.8–2.30
Number of reflections	13,402	29,867
*R*_work_/*R*_free_ (%)	21.4/24.8	20.7/24.7
**No. of atoms**		
Protein (chain A/B)	6220	3079/3050
Zn^2+^	2	–
SO_4_^2-^	50	–
Water (chain A/B)	6	20/18
Other (chain A/B)	23	24/24
**Average B factors**		
Protein (Å^2^)	91	62
Water (Å^2^)	67	81
Other (Å^2^)	111	92
**r.m.s. deviations**		
Bond length (Å)	0.013	0.0325
Bond angles (°)	1.66	2.32
**Ramachandran plot**		
Favoured (%)	97	98
Allowed (%)	3	2
Disallowed (%)	0	0

^a^Values in the parentheses denote the highest resolution shell. R_free_ was calculated using a test set comprising 5% of the data.

^b^Calculated using the MOLPROBITY program^72^.

Crystals of Zn-free MsBP diffracted to 2 Å, but data used for model building and refinement was trimmed to 2.3 Å. Coordinates of Zn-MsBP (6WPM) were used to phase the native MsBP dataset using molecular replacement (Phaser), although the latter form contained two chains per a.s.u. Refinement was initiated with the full chain of 410 residues, but following rounds of manual building (*Coot*), density modification (Parrot)^62^, and automated refinement (RefMac)^63^, polypeptide segments in areas of poor density were progressively removed. The structure of MsBP is nearly identical to the experimentally phased Zn-MsBP**.** However, we note that despite a lower overall B-factor (average 50 Å^2^), chain breaks are found across both monomers (Chain A: L18-P20, P48-S51, A108-E112; and Chain B: P48-S51, L139-A142, G271-G274, and V363-A365), corresponding to dynamic loop regions of indistinguishable electron density. The structure of MsBP contains one Ramachandran outlier (Chain A: R192). Overall, MsBP also has a low solvent content, with 38 ordered waters distinguishable across both chains. A final R_free_ value of 0.20 was obtained for this data, with only 0.5% of sidechains in outlier conformations. Final coordinates of Zn-free MsBP are deposited in the PDB (6WPN). Final models were assessed using the wwPDB validation server^64^, and structural homologues were identified using DALI^65^ as of September 2021.

### Molecular docking

Ligand docking was carried out using the AutoDock Vina program^66^. The structure of Zn-MsBP (6WPM) was prepared by removing water and sulphate molecules. Hydrogen atoms were added, and charges (Kollman) assigned using MGL Tools (https://ccsb.scripps.edu/mgltools/). The grid area for ligand docking was defined from the binding cleft. Grid dimensions were 30 × 32 × 30 points centred on (*x,y,z)* coordinates (58.799, 25.510, 24.135). The chemical structure of fructose-1,6-bisphosphate (FBP, PubChem CID: 445557) was downloaded from PubChem^67^. FBP was docked within the defined grid space using the default AutoDock Vina parameters (grid dimensions as provided; energy range = 4; exhaustiveness = 8). Each conformer was manually inspected using PyMOL to assess the plausibility of the fit and to predict potential interacting sidechains. As the grid search area was larger than 2700 Å^2^, the exhaustiveness was then increased until convergence was reached (exhaustiveness = 100).

### MsBP mutant design

Single-site mutants were prepared using the QuikChange Multi Site-Directed Mutagenesis kit (Agilent). Primers were designed and mutant DNA generated using the PCR cycle and reagent concentrations recommended by the manufacturer, with the addition of 0.5 μL of the optional QuikSolution per 25 μL reaction. Following amplification, the reaction was cooled on ice (2 min) and then treated with DpnI (10 U per reaction) and incubated at 37 °C for 1 h. The digested reaction mixture was transformed into XL10-Gold ultracompetent cells treated with β-mercaptoethanol, following the manufacturer’s instructions. Mutants were verified by sequencing before further purification and characterisation.

## Supplementary Information

Supplementary Information.

## Data Availability

Coordinates and structure factors for crystal structures presented in this article have been deposited in the RSCB Protein Data Bank (https://www.rcsb.org) under the following accession codes: 6WPM (Zn-MsBP); https://www.rcsb.org/structure/6WPM, 6WPN (MsBP); https://www.rcsb.org/structure/6WPN.

## Abbreviations

SBP: Substrate-binding protein
MsBP: Modified sugar binding protein
a.s.u: Asymmetric unit
G6P: Glucose-6-phosphate
FBP: Fructose-1,6-bisphosphate
DexS: Dextran sulphate
T_*M*_: Melting temperature
DSF: Differential scanning fluorimetry
ITC: Isothermal titration calorimetry
Zn: Zinc
SO_4_: Sulphate
