# Supplementary material for: Novel functional insights into a modified sugar-binding protein from Synechococcus MITS9220
Source: Sci Rep. 2022 Mar 21;12:4805. doi: 10.1038/s41598-022-08459-8 (PMC8938411; doi:10.1038/s41598-022-08459-8)
Supplement: Supplementary file 1 — Supplementary Information. [file 41598_2022_8459_MOESM1_ESM.pdf]

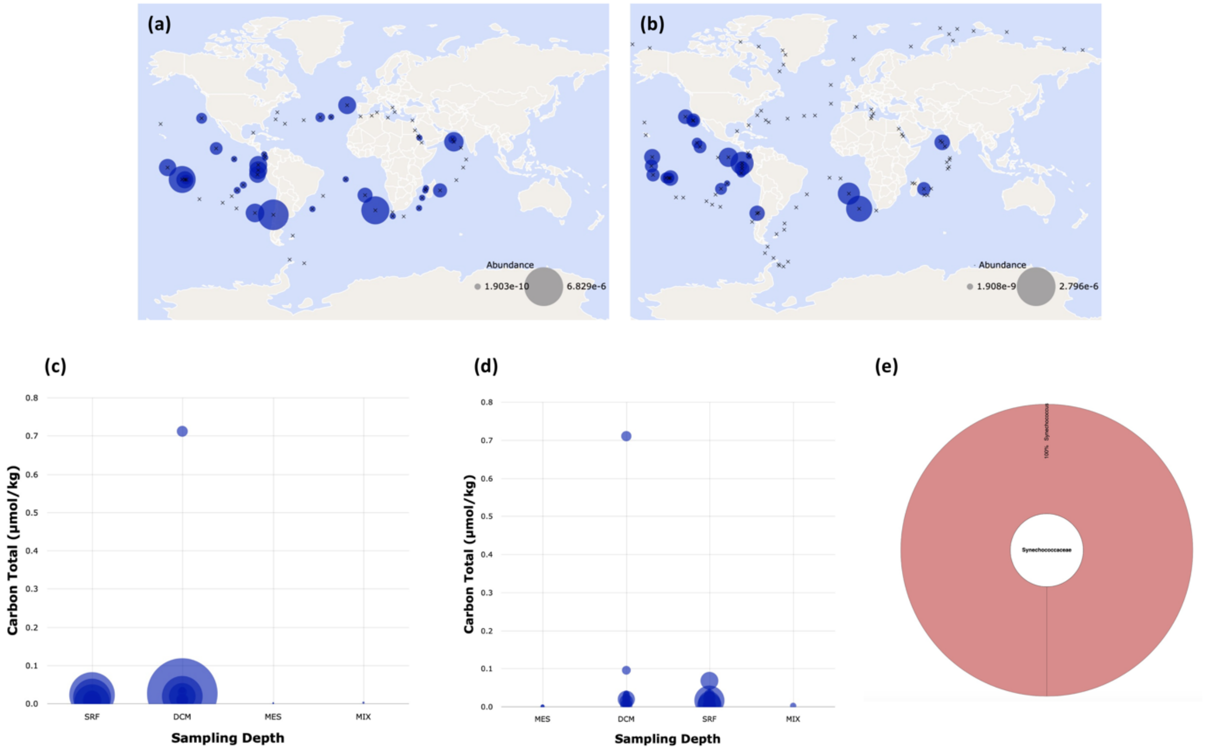

Supporting Information Figure S1: Gene abundance data for MsBP and its homologs. The normalised abundance of MsBP from *Synechococcus* MITS9220 was plotted using the publicly-available *Tara* Oceans data [25] through the Ocean Gene Atlas tool [26]. The nucleotide sequence of the *MsBP* gene from MITS9220 was used to search the Atlas with the default parameters (threshold 1E-10). The gene abundance of *MsBP* and its homologues (blue circles) from Tara Oceans metagenomes **(a)** and Tara Oceans metatranscriptomes **(b)** for each sampling site (denoted with an X) at various depth was assessed, indicating this gene to predominately be found in the surface and deep chlorophyll maximum (DCM) layers. Bubble plots of the gene abundance as a function of total carbon across all sampling depths is depicted for the Tara Oceans metagenomes **(c)** and Tara Oceans metatranscriptomes **(d).** The taxonomic breakdown of homologues identified **(e)** show the gene is exclusively present in *Synechococcus* strains.

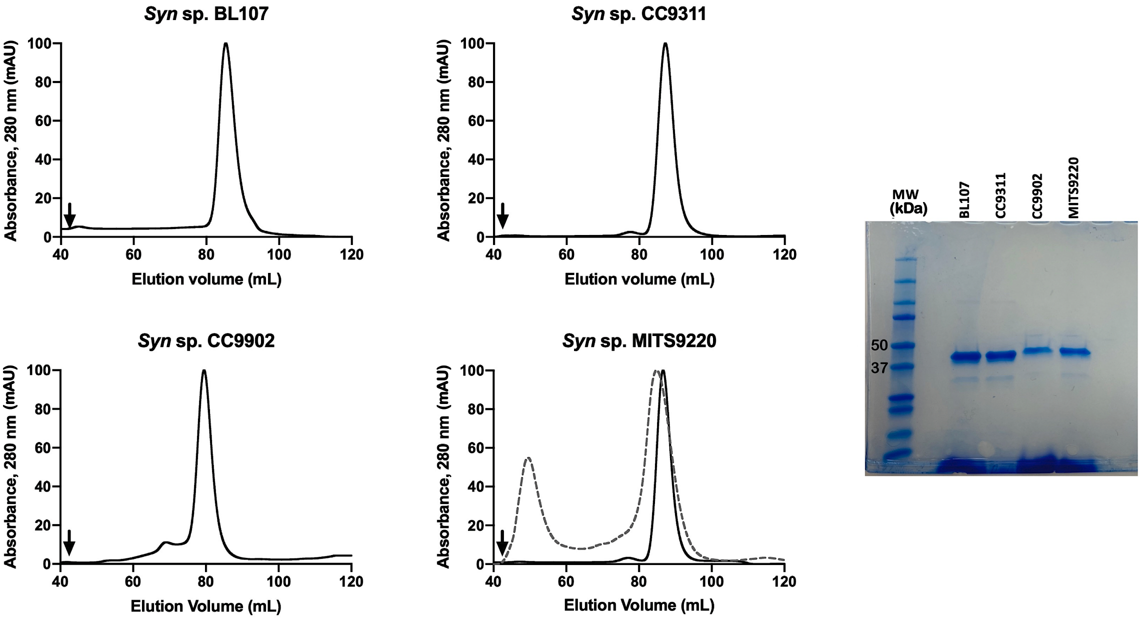

Supporting Information Figure S2: Purification of recombinant MsBP obtained from different *Synechococcus* isolates. His-tagged MsBP originating from four different *Synechococcus* isolates (BL107, CC9311, CC9902, and MITS9220) was purified from large-scale cultures of *E. coli*. Chromatography traces are indicated for each purified protein showing each to be a monodisperse product. Analysis by SDS-PAGE confirms an apparent molecular weight consistent with that expected based on comparison to standards of known size (BioRad Precision Plus Dual Colour) and >95 % purity for each product prior to subsequent analysis.

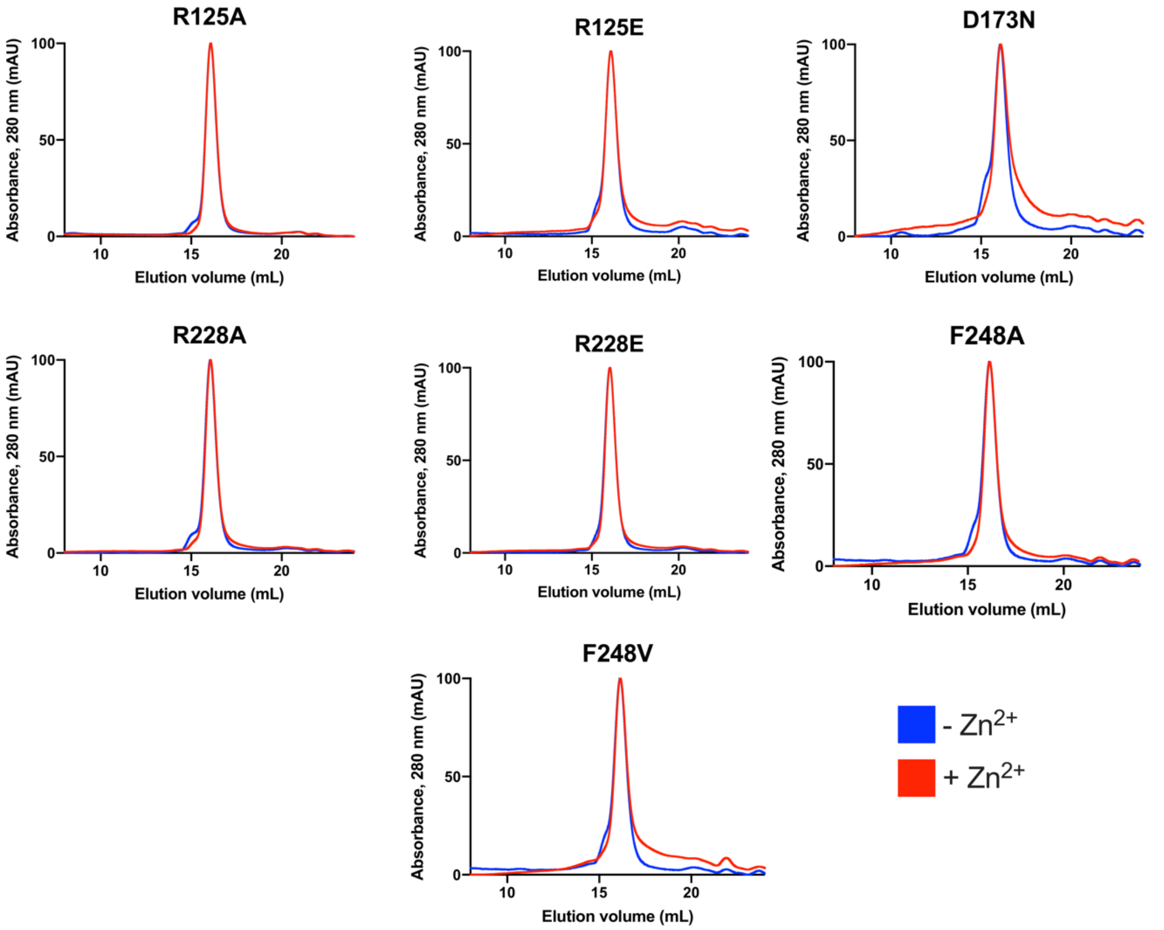

Supporting Information Figure S3: Size-exclusion chromatography elution profiles for MsBP single-site mutants. Single-site mutants were designed to probe the effect of identified residues on zinc-mediated avidity for ligand analogues, such as cross-linked dextran sulphate used for size-exclusion analysis. The chromatographic traces are shown for each mutant both in the absence (blue) and presence (red) of zinc. Compared to the native MsBP (**Fig. 2**), no mutants showed altered elution profiles upon the addition of zinc.

Supporting Information Table S1: Sugar compounds tested during ligand screening

Compound	Class	Compound	Class	Compound	Class	Compound	Class
α-D-glucose	aldohexose	sedoheptulose	ketoheptose	2-deoxy adenosine	deoxynucleoside	uridine-3',5'-cyclic monophosphate	nucleoside
β-D-allose	aldohexose	D-psicose	ketohexose	chondroitin sulphate C	glycosaminoglycan	uridine-5'-monophosphate	nucleoside
D-galactose	aldohexose	D-fructose	ketonic monosaccharide	D-glucosamine	hexosamine	2-deoxy-D-glucose-6-phosphate	phosphosugar
D-mannose	aldohexose	L-sorbose	ketose	N-acetyl-β-D-mannosamine	hexosamine	6-phosphogluconic acid	phosphosugar
L-glucose	aldohexose	glycerol	polyol	N-acetyl-D-glucosamine	hexosamine	D-fructose-6-phosphate	phosphosugar
D-arabinose	aldopentose	dextrin	polysaccharide	glucuronamide	hexose derivative	D-glucosamine-6-phosphate	phosphosugar
D-ribose	aldopentose	inulin	polysaccharide	adenosine	nucleoside	D-glucose-1-phosphate	phosphosugar
D-xylose	aldopentose	laminarin	polysaccharide	adenosine 3',5'-cyclic monophosphate	nucleoside	D-glucose-6-phosphate	phosphosugar
L-arabinose	aldopentose	mannan	polysaccharide	adenosine-2'-monophosphate	nucleoside	D-mannose-1-phosphate	phosphosugar
L-lyxose	aldopentose	pectin	polysaccharide	adenosine-2',3'-cyclic monophosphate	nucleoside	D-mannose-6-phosphate	phosphosugar
N-acetyl-D-neuraminic acid	amino sugar	gelatin	protein polymer	adenosine-3'-monophosphate	nucleoside	Fructose-1,6-bisphosphate	phosphosugar
glycogen	branched polysaccharide	β-methyl-D-glucuronic acid	sugar acid	adenosine-5'-monophosphate	nucleoside	inositol hexaphosphate	phosphosugar
m-inositol	carbocyclic sugar	D-galacturonic acid	sugar acid	cytidine-2'-monophosphate	nucleoside	3-methyl glucose	sugar derivative
α-cyclodextrin	cyclic oligosaccharide	D-gluconic acid	sugar acid	cytidine-2',3'-cyclic monophosphate	nucleoside	α-methyl-D-galactoside	sugar derivative
β-cyclodextrin	cyclic oligosaccharide	D-glucosaminic acid	sugar acid	cytidine-3'-monophosphate	nucleoside	α-methyl-D-glucoside	sugar derivative
γ-cyclodextrin	cyclic oligosaccharide	D-glucuronic acid	sugar acid	cytidine-3',5'-cyclic monophosphate	nucleoside	α-methyl-D-mannoside	sugar derivative
D-fucose	deoxyhexose	D-saccharic acid	sugar acid	cytidine-5'-monophosphate	nucleoside	β-methyl-D-galactoside	sugar derivative

L-fucose	deoxyhexose	adonitol	sugar alcohol	guanosine-2'-monophosphate	nucleoside	β-methyl-D-glucoside	sugar derivative
L-rhamnose	deoxyhexose	D-arabitol	sugar alcohol	guanosine-2',3'-cyclic monophosphate	nucleoside	β-methyl-D-xyloside	sugar derivative
2-deoxy-D-ribose	deoxypentose	D-mannitol	sugar alcohol	guanosine-3'-monophosphate	nucleoside	1-thio-β-D-glucose	sulphosugar
3-0-B-D-galactopyranosyl-D-arabinose	disaccharide	D-sorbitol	sugar alcohol	guanosine-3',5'-cyclic monophosphate	nucleoside	Dextran sulphate	sulphosugar
α-D-lactose	disaccharide	dulcitol	sugar alcohol	guanosine-5'-monophosphate	nucleoside	D-tagatose	hexose
D-cellobiose	disaccharide	i-erythritol	sugar alcohol	Inosine	nucleoside	glucuronamide	hexose derivative
D-melibiose	disaccharide	L-arabitol	sugar alcohol	thymidine	nucleoside		
D-trehalose	disaccharide	lactitol	sugar alcohol	thymidine-3'-monophosphate	nucleoside		
gentiobiose	disaccharide	maltitol	sugar alcohol	thymidine-3',5'-cyclic monophosphate	nucleoside		
lactulose	disaccharide	N-acetyl-D-glucosaminitol	sugar alcohol	thymidine-5'-monophosphate	nucleoside		
maltose	disaccharide	xylitol	sugar alcohol	uridine	nucleoside		
melibionic acid	disaccharide	stachyose	tetrasaccharide	uridine-2'-monophosphate	nucleoside		
palatinose	disaccharide	D-melezitose	trisaccharide	uridine-2',3'-cyclic monophosphate	nucleoside		
sucrose	disaccharide	D-raffinose	trisaccharide	uridine-3'-monophosphate	nucleoside		
turanose	disaccharide	maltotriose	trisaccharide				

Supporting Information Table S2: Analysis of hinge bending and domain closure.

Protein	Unliganded conformation	Liganded conformation	Ligand chain length /monomer units	Rotation angle /°	Closure /%
GacH	3K01	3K00	4	29.5	99.8
		3K02	4	29.5	99.7
		3JZJ	4	29.4	99.7
SO-BP	5YSB	5YSD	3	25.4	98.8
		5YSE	4	26.1	97.5
		5YSF	5	24.5	98.8
TmMBP	6DTR	6DTQ	2	35.8	100
		6DTS	4	51.2	99.4
		6DTU	4	56.1	99.8

Table S3: Ligand docking with Zn-MsBP

Ligand	Rank	Predicted binding energy (kcal mol^{-1})	Dist. from best (r.m.s.d.)	
			Lower bound	Upper bound
	1	-6.6	0	0
	2	-6.5	2.345	4.368
	3	-6.5	1.140	5.254
	4	-6.4	2.079	4.169
**FBP**	5	-6.4	2.037	4.034
	6	-6.3	2.400	4.536
	7	-6.2	9.840	11.821
	8	-6.2	2.211	4.143
	9	-6.2	1.272	5.650
